# Intermittent Fasting Improves High-Fat Diet-Induced Obesity Cardiomyopathy via Alleviating Lipid Deposition and Apoptosis and Decreasing m6A Methylation in the Heart

**DOI:** 10.3390/nu14020251

**Published:** 2022-01-07

**Authors:** Zujie Xu, Ying Qin, Binbin Lv, Zhenjun Tian, Bing Zhang

**Affiliations:** 1 Department of Physical Education, Tsinghua University, Beijing 100081, China; xuzj20@mails.tsinghua.edu.cn (Z.X.); qin-y20@mails.tsinghua.edu.cn (Y.Q.); lbb20@mails.tsinghua.edu.cn (B.L.); 2 Institute of Sports Biology, College of Physical Education, Shaanxi Normal University, Xi’an 710119, China

**Keywords:** intermittent fasting, high-fat diet, N6-methyladenosine methylation, obesity cardiomyopathy, lipid deposition, apoptosis

## Abstract

Intermittent fasting (IF) plays an essential role in improving lipid metabolism disorders caused by metabolic cardiomyopathy. Growing evidence revealed that N6-methyladenosine (m6A) RNA methylation is related to obesity and lipid metabolic. Our study aimed to assess the beneficial effects of IF on lipid deposition, apoptosis, and m6A methylation in high-fat diet (HFD)-induced obesity cardiomyopathy. Male C57BL/6J mice were fed a normal diet (ND) or HFD ad libitum for 13 weeks, after which time a subgroup of HFD mice were subjected to IF for 24 h and fed HFD in the other day for 8 weeks. We found that IF intervention significantly improved cardiac functional and structural impairment and serum lipid metabolic disorder induced by HFD. Furthermore, IF intervention decreased the mRNA levels of the fatty acid uptake genes of FABP1, FATP1, and CD36 and the fatty acid synthesis genes of SREBF1, FAS, and ACCα and increased the mRNA levels of the fatty acid catabolism genes of ATGL, HSL, LAL, and LPL in cardiac tissueof HFD-induced obese mice. TUNEL-positive cells, Bax/Bcl-2 ratio, and Cleaved Caspase-3 protein expression in HFD-induced obese mice hearts was down-regulated by IF intervention. In addition, IF intervention decreased the m6A methylation levels and METTL3 expression and increased FTO expression in HFD-induced obesity cardiomyopathy. In conclusion, our findings demonstrate that IF attenuated cardiac lipid deposition and apoptosis, as well as improved cardiac functional and structural impairment in HFD-induced obesity cardiomyopathy, by a mechanism associated with decreased m6A RNA methylation levels.

## 1. Introduction

Obesity cardiomyopathy is defined as obesity-induced impairment in the cardiac architecture and function that is independent of hypertension, coronary heart disease, and other heart diseases [1,2]. High-fat diet (HFD)-induced obesity cardiomyopathy is characterized by abnormal heart structure and dysfunction, such as echocardiographic changes consistent with poor systolic function, enhanced cardiac lipid deposition, and apoptosis [3,4,5]. Clinical and experimental evidence has demonstrated that myocardial lipid metabolic disorder is the initial cellular pathogenesis of obesity cardiomyopathy, which causes cardiomyocyte injury by triggering apoptosis [6,7,8]. Hence, further carrying out effective research on the regulation of myocardial lipid deposition is necessary and could provide new insights into potential therapeutic approaches for obesity cardiomyopathy.

Intermittent fasting (IF), a nutritional approach in which ad libitum feeding is alternated with fasting periods, has been shown to have cardioprotective effects in the heart [9,10]. Both clinical and experimental evidence has revealed that IF extends lifespan, decreases myocardial triglyceride accumulation, inhibits cardiac cell apoptosis, improves cardiac diastolic parameters, and activates a cardioprotective metabolic program [11,12,13]. Furthermore, growing evidence showed that IF participates in attenuating abnormal lipid metabolism [14,15]. IF ameliorates HFD or high-fructose diet-induced myocardial injury by modulating the left ventricular renin-angiotensin system [16]. In addition, IF exerts beneficial lipid metabolic effects by improving gut microbiota in HFD-induced obese mice [17]. However, the underlying mechanism by which IF regulates lipid metabolism remains unknown.

N6-methyladenosine (m6A) methylation, the most common and abundant epigenetic modification of eukaryotic mRNA, is catalyzed by m6A methyltransferase, or writers ( methyltransferase like 3/14 (METTL3/14) and Wilms tumor 1-associated protein (WTAP)), removed by m6A demethylating enzymes, or erasers ( fat mass and obesity-associated protein (FTO) and α-ketoglutarate-dependent homolog 5 (ALKBH5)), and recognized by m6A-binding proteins, or readers (YTH domain-containing family 1/2/3 (YTHDF1/2/3) and YTH domain-containing protein 1/2 (YTHDC1/2)) [18]. Modification of m6A on mRNA functionally affects multiple RNA processes, including stability, splicing, translation, and degradation [19]. Recent studies show m6A methylation is related to obesity, lipid metabolism, and apoptosis, and plays an essential role in the physiological and pathological processes of cardiovascular diseases [20,21,22]. However, the effect of IF on m6A methylation modification remains unclear.

We assumed that IF in a HFD improved myocardial lipid deposition and apoptosis due to changes in m6A modification levels, thereby ameliorating obesity cardiomyopathy. Therefore, our study was designed to assess the effects of IF on lipid deposition, apoptosis, and m6A methylation in cardiac tissueof obese mice.

## 2. Materials and Methods

### 2.1. Animal and Diets

All animal experiments in our study were reviewed and approved by the Institutional Animal Care and Use Committee of Tsinghua University (identification number: F16-00228; A5061-01). Three- to four-week-old male C57BL/6J mice were purchased from the Laboratory Animal Research Center of Tsinghua University and bred in the specific pathogen-free experimental animal environment at the Laboratory Animal Research Center of Tsinghua University, with five mice per cage and a 12 h light/12 h dark cycle in a temperature-controlled environment.

**Figure 1:**
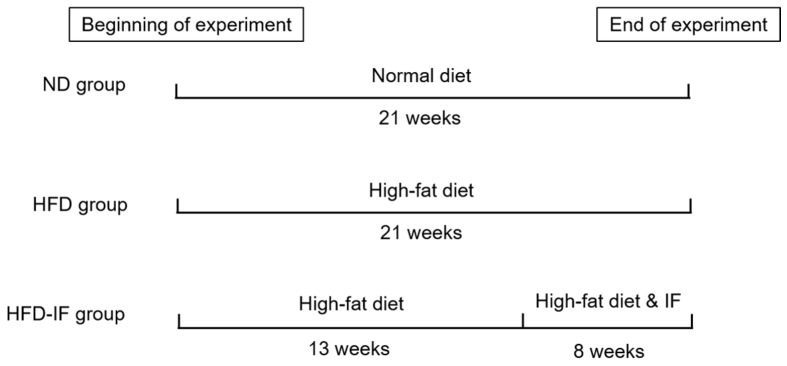
Schematic diagram of experimental design. Normal diet (ND) group: mice were fed ND for 21 weeks. High-fat diet (HFD) group: mice were fed HFD for 21 weeks. HFD-IF group: mice were fed HFD for 13 weeks and subjected to intermittent fasting (IF) for 24 h and fed HFD in the other day for 8 weeks.

After 1 week of acclimation, the mice were randomly divided into 2 groups: normal diet (ND, *n* = 15) and HFD (*n* = 30). ND has 20% kcal from protein, 70% kcal from carbohydrates, and 10% kcal from fat, and HFD has 20% kcal from protein, 20% kcal from carbohydrates, and 60% kcal from fat (Beijing Keao Xieli Feed Co., Ltd., Beijing, China). After 13 weeks of being fed HFD, each of the 30 mice gained 20% more body weight than at week 0. Therefore, it can be considered that the obesity model was established. The mice fed HFD were further divided into 2 groups to continue having ad libitum access to HFD or to have IF access to food (HFD-IF, *n* = 15) for 8 weeks. Mice in the HFD-IF group were allowed free access to regular chow every other day and no food on the alternate day [23]. No mice were excluded during the 21-week experiment. The experimental design is shown in Figure 1. The body weight and food consumption were recorded weekly. At the end of the experiment, the eyeball blood of mice was taken after fasting for 12 h. The myocardial tissues were rapidly removed, washed with cold normal saline, dried by blotting on filter paper, weighed, and stored at −80 °C for later use.

### 2.2. Echocardiography

The cardiac physiological functions were assessed in anesthetized (isoflurane) mice using a two-dimensional guided M-mode echocardiography (VINNO 6 VET, VINNO, Suzhou, China) [24]. The left ventricle internal dimension diastole (LVIDd), left ventricle internal dimension systole (LVIDs), and ejection fraction (EF) were evaluated by M-mode echocardiography. Fractional shortening (FS) was calculated with the following formula: FS = (LVIDd − LVIDs)/LVIDd × 100%.

### 2.3. Biochemical Parameters

Serum levels of glucose (GLU), total cholesterol (TC), triglycerides (TG), high-density lipoprotein (HDL), and low-density lipoprotein (LDL) were measured with a biochemical analyzer (KHB-ZY 1280, Shanghai Kehua Bio-engineering Co., Ltd., Shanghai, China). Free fatty acid (FFA) contents in serum were determined by an ELISA kit (ab65341, Abcam, Cambridge, MA, USA) according to the manufacturer’s protocols. Levels of TG in myocardial tissue were measured using a commercially available kit (Nanjing Jiancheng Bioengineering Institute, Nanjing, China).

### 2.4. m6A RNA Methylation Quantification

An EpiQuik m6A RNA methylation quantification kit (EpiGentek, Wuhan, China) was used to quantify the global RNA m6A content in cardiac tissue samples. In brief, add 200 ng of total RNA to the well, incubate with the capture antibody for 1 h, then incubate with the detection antibody for 30 min, and then incubate with the enhancer solution for 30 min at −37 °C. Quantification was measured by reading the absorbance at 450 nm using a microplate spectrophotometer. The m6A levels in the total RNA was calculated by OD intensity.

### 2.5. Histology and Oil Red O Staining

The myocardial tissues were fixed in 4% paraformaldehyde overnight and embedded in paraffin. For the detection of morphological changes, 5-μm-thick dewaxed sections were stained with hematoxylin and eosin (H&E). The heart sections were stained with standard Masson trichrome staining to estimate interstitial fibrosis. To evaluate cardiac lipid accumulation, the frozen myocardial tissue was cut in Tissue-Tek OCT (Sakura-Finetek, Tokyo, Japan), and then the heart sections (10 μm) were stained with Oil Red O (Solarbio, Beijing, China). Histopathology images were observed and acquired with an Olympus optical microscope (Olympus, Tokyo, Japan).

### 2.6. Transmission Electron Microscopy (TEM)

The mice left ventricle was cut into 1 mm^3^ piece and fixed with 2.5% glutaraldehyde in 0.1 M sodium phosphate (pH 7.4) for 24 h at 4 °C. The heart tissue samples were embedded, cut, and stained with uranyl acetate and lead citrate. Lipid droplets (LDs) were observed by TEM (H-7650B, Ibaraki, Hitachi, Japan).

### 2.7. TUNEL Staining

Apoptosis was examined via using terminal deoxynucleotidyl transferase-mediated dUTP nick end-labeling (TUNEL) staining according to the manufacturer’s protocols (Beyotime, Nanjing, China). 5μm heart sections were incubated with a TUNEL reagent mixture and incubated in dark at 37 °C for 1 hand then rinsed three times for 5 min each with PBS. The green positive particles were visualized under a Nikon fluorescence microscope (Nikon, Tokyo, Japan). 

### 2.8. Quantitative Real-Time PCR (RT-PCR)

Total RNA of mouse cardiac was extracted using Trizol reagent. Reverse transcription was performed with the PrimeScript TM RT-PCR kit (TaKaRa, Tokyo, Japan). RT-PCR was detectedusing Quantitative PCR with SYBR Green PCR Master Mix (Beyotime, Nanjing, China) and CFX96 Real-Time PCR System (Bio-Rad, Hercules, CA, USA). The quantitative data were obtained with the 2^−ΔΔCt^ method and normalized to GAPDH. RT-PCR analysis was performed for fatty acid binding protein 1 (FABP1), fatty acid transporter 1 (FATP1), cluster of differentiation 36 (CD36), sterol regulatory element-binding factor 1 (SREBF1), fatty acid synthase (FAS), acetyl- CoA carboxylase α (ACCα), adipose triglyceride lipase (ATGL), lysosomal acid lipase (LAL), hormone-sensitive lipase (HSL), lipoprotein lipase (LPL), METTL3, METTL14, WTAP, FTO, ALKBH5, YTHDF1, YTHDF2, YTHDF3, YTHDC1, YTHDC2, and GAPDH. All primers were produced by Sangon (Shanghai, China), and the primers used in this study are shown in Table 1.

### 2.9. Western Blot

Total protein was isolated from heart tissue with a RIPA buffer, and the protein concentration was measured with a BCA assay kit. The protein samples were separated by SDS-polyacrylamide gels with suitable concentration and then transferred onto polyvinylidene fluoride (PVDF) membranes. The membranes were blocked with 5% bull serum albumin and then developed with diluted antibodies for Bax (14796, 1:1000, Cell Signaling Technology, Beverly, MA, USA), Bcl-2 (BS1511, 1:1000, Bioworld, Bloomington, USA), Cleaved Caspase-3 (9661, 1:1000, Cell Signaling Technology, Beverly, MA, USA), METTL3 (ab240595, 1:1000, Abcam, Cambridge, MA, USA), FTO (27226-1-AP, 1:1000, Proteintech, Wuhan, China), YTHDF1 (17479–1-AP, 1:1000, Proteintech, Wuhan, China), and GAPDH (10494-1-AP, 1:5000, Proteintech, Wuhan, China) overnight at 4 °C. Subsequently, membranes were incubated with horseradish peroxidase- (HRP-) conjugated secondary antibody at room temperature for 90 min. The membranes were observed on Image Systems (Bio-Rad, Hercules, CA, USA). Image Lab software was used for semiquantitative calculation.

### 2.10. Statistical Analysis

Results were shown as the mean ± standard error of the mean (SEM) from at least three independent experiments. Statistical differences between the groups were analyzed using One-way analysis of variance (ANOVA) and post hoc least significant difference (LSD) multiple-comparison test. *p* < 0.05 was considered statistically significant (* *p* < 0.05, ** *p* < 0.01 are indicated in figures).

## 3. Results

### 3.1. IF Improves HFD-Induced Mice Obesity Cardiomyopathy

**Figure 2:**
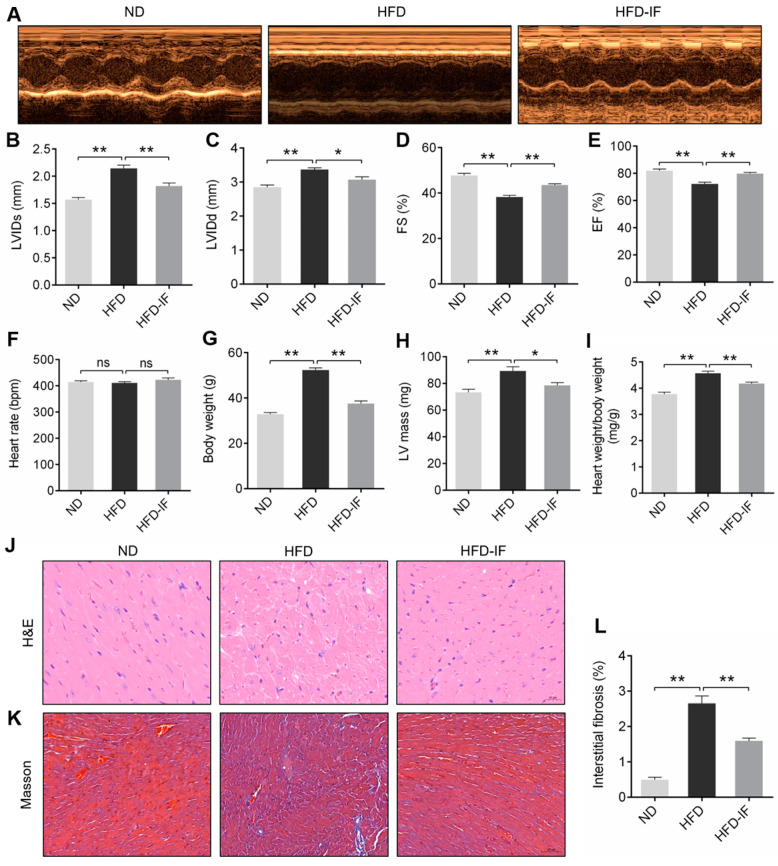
Effects of IF on myocardial structure and function in HFD-fed mice. (**A**) Echocardiographic measurements of (**B**) LVIDs, (**C**) LVIDd, (**D**) FS%, and (**E**) EF% for cardiac functional analysis. Measurements of (**F**) heart rate, (**G**) body weight, (**H**) LV mass, and (**I**) heart weight/body weight. Representative images of (**J**) H&E staining (scale bar = 20 μm) and (**K**) Masson staining (scale bar = 50 μm) cardiac sections. (**L**) Quantitative analysis of interstitial fibrosis. Data are shown as means ± SEM. * *p* < 0.05, ** *p* < 0.01. ns: no significant difference.

Echocardiography was performed in HFD mice to determine whether IF had an improvement effect on cardiac function. Compared with mice fed with ND, 21-week HFD significantly decreased FS% and EF% and increased LVIDs and LVIDd. These changes caused by HFD were markedly restored in the hearts treated with IF (Figure 2A–E). HFD and HFD-IF did not significantly affect heart rate (Figure 2F). Furthermore, HFD feeding significantly caused a higher body weight, LV mass, and heart-to-body weight ratio, which were significantly decreased by IF intervention (Figure 2G–I). H&E and Masson staining indicated that morphological abnormalities and interstitial fibrosis were observed in the myocardial tissues of obese mice; however, the above damage was remarkably reversed in HFD-IF groups (Figure 2J–L). Together, these results demonstrated that IF showed a promising approach in improved obesity cardiomyopathy induced by HFD, suggesting the protective effects on cardiomyocyte injury and dysfunction in obese mice.

### 3.2. IF Ameliorates HFD-Induced Serum Lipid Metabolic Disorder

**Figure 3:**
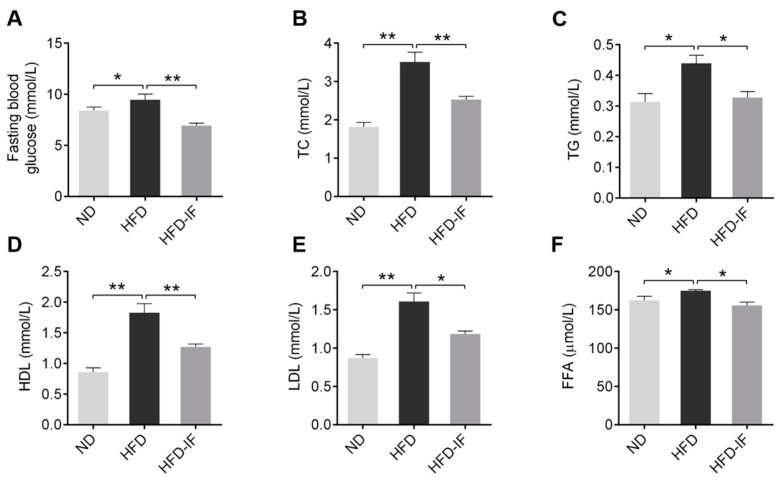
IF improves HFD-induced adverse lipid metabolism in serum. (**A**) Fasting blood glucose, (**B**) TC, (**C**) TG, (**D**) HDL, (**E**) LDL, and (**F**) FFA were measured in serum. Data are shown as means ± SEM. * *p* < 0.05, ** *p* < 0.01.

In comparison to the NC group, HFD significantly increased in serum fasting blood glucose, TC, TG, HDL, LDL, and FFA; however, these effects were remarkably mitigated in the HFD-IF groups (Figure 3A–F). It was indicated that IF could attenuate serum lipid metabolic disorder induced by HFD.

### 3.3. IF Alleviates HFD-Induced Cardiac Lipid Deposition

**Figure 4:**
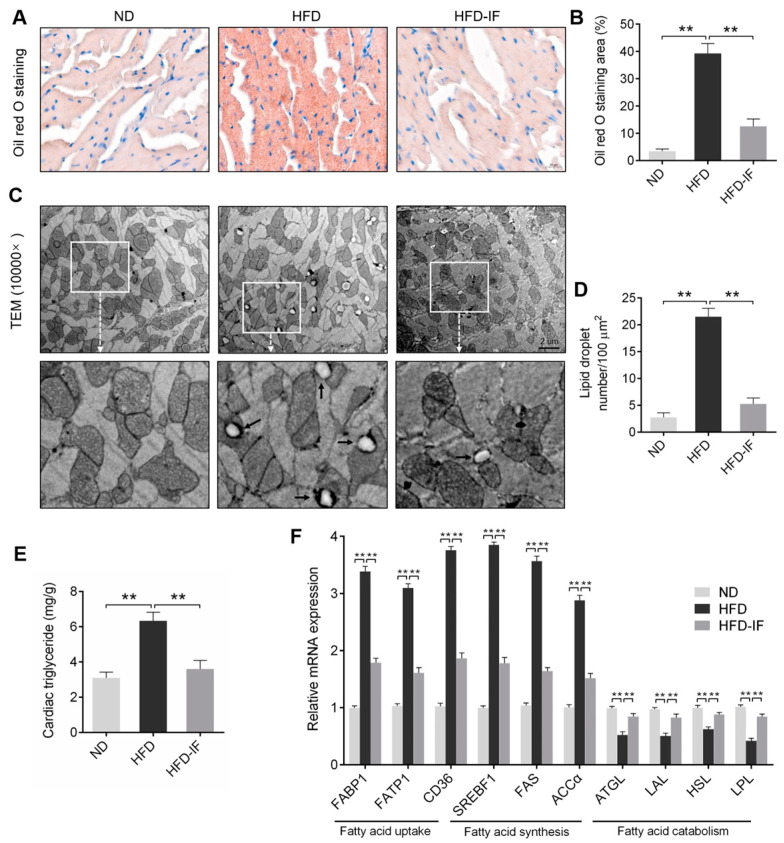
IF improves cardiac lipid deposition in HFD-fed mice. (**A**) Representative images and (**B**) quantitative analysis of Oil Red O staining heart sections. Scale bar = 20 μm. (**C**) LDs were observed by TEM at 10,000× magnification. Scale bar = 2 μm. The lower image is an amplification of the upper white box. LDs were marked with black arrows. (**D**) Quantitative analysis of TEM at 10,000×. (**E**) Levels of TG in heart of mice. (**F**) RT-PCR analysis of the mRNA expression of genes regulating fatty acid uptake (FABP1, FATP1, and CD36), fatty acid synthesis (SREBF1, FAS, and ACCα), and fatty acid catabolism (ATGL, LAL, HSL, and LPL) in cardiac tissues. Data are shown as means ± SEM. ** *p* < 0.01.

Metabolic perturbations stemming from obesity could lead to lipid deposition in myocardium [25]. Oil Red O staining and TEM suggested that HFD significantly increased lipid deposition and LDs accumulation in hearts; these abnormalities were restored in the HFD-IF groups (Figure 4A–D). Additionally, HFD-induced lipid deposition increases the levels of TG in cardiac were down-regulated by IF intervention (Figure 4E). What’s more, long-term HFD significantly increased the mRNA expression of genes regulating fatty acid uptake (FABP1, FATP1, and CD36) and fatty acid synthesis (SREBF1, FAS, and ACCα), and reduced the mRNA expression of genes regulating fatty acid catabolism (ATGL, LAL, HSL, and LPL); but these effects were obviously restrained by IF intervention (Figure 4F). Collectively, these findings suggested that IF was effective in reducing cardiac lipid deposition in obese mice.

### 3.4. IF Inhibites HFD-Induced Cardiac Apoptosis

**Figure 5:**
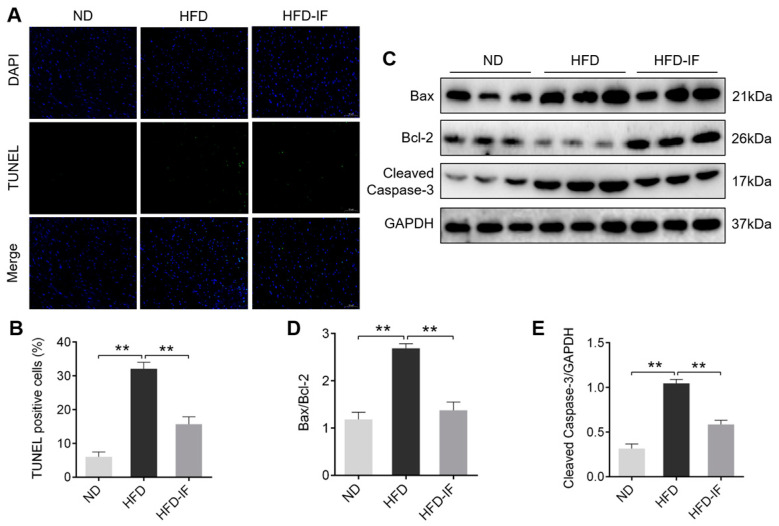
IF inhibits cardiac apoptosis in HFD-fed mice. (**A**) Representative images of TUNEL staining cardiac sections. The positive apoptotic particles in green and DAPI in blue. Scale bar = 50 μm. (**B**) Quantitative analysis of TUNEL-positive cells. (**C**–**E**) Western blot images and their densitometric quantitative analysis of Bax/Bcl-2 ratio and Cleaved Caspase-3. Data are shown as means ± SEM. ** *p* < 0.01.

Ectopic deposition of myocardial lipids could lead to apoptosis in the heart [26,27]. TUNEL staining revealed that HFD-induced positive apoptotic particles were downregulated by IF intervention (Figure 5A,B). Consistently, western blot analysis showed that Bax/Bcl-2 ratio and Cleaved Caspase-3 protein expression were significantly increased in the HFD group compared to the ND group. However, IF significantly restored these effects (Figure 5C–E). These results demonstrated that obesity-induced apoptosis in cardiac tissues could be inhibited by IF intervention.

### 3.5. IF Decreases HFD-Induced Cardiac m6A Methylation

**Figure 6:**
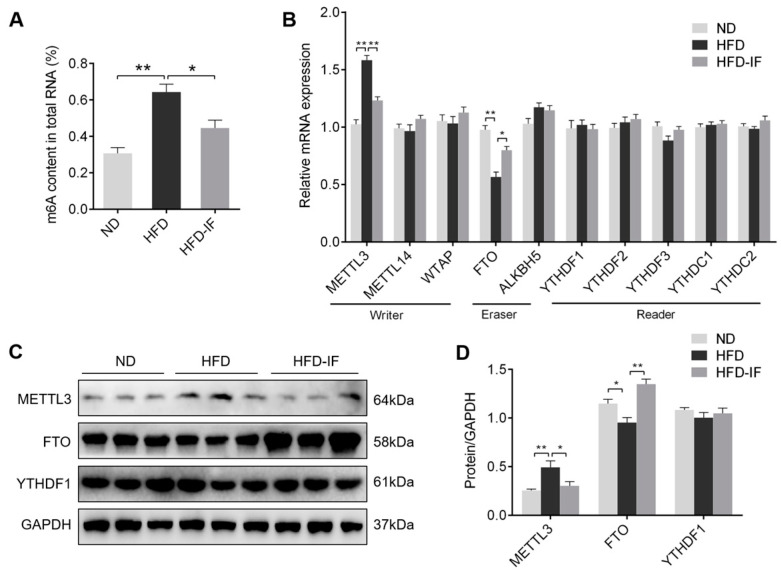
IF decreases cardiac m6A methylation in HFD-fed mice. (**A**) The m6A level in heart tissue samples. (**B**) RT-PCR analysis of the mRNA levels of m6A methylation-related genes in cardiac tissues related to writer (METTL3, METTL14, and WTAP), eraser (FTO and ALKBH5), and reader (YTHDF1, YTHDF2, YTHDF3, YTHDC1, and YTHDC2). (**C**,**D**) Western blot images and their densitometric quantitative analysis of METTL3, FTO, and YTHDF1. Data are shown as means ± SEM. * *p* < 0.05, ** *p* < 0.01.

To assess the effect of IF on m6A methylation, we measured m6A content and m6A-associated genes and proteins in cardiac tissues. IF intervention markedly rescued m6A content in the heart from HFD-fed mice (Figure 6A). Additionally, the gene expression of METTL3 was up-regulated by HFD, and the FTO mRNA levels was down-regulated; however, IF intervention markedly reversed HFD-induced these effects, which were also confirmed by western blot (Figure 6B–D). Taken together, these results indicated that IF intervention decreases cardiac m6A methylation levels in HFD-fed mice.

## 4. Discussion

As we know, cardiac tissueis the major attacking target of obesity. HFD-induced obesity cardiomyopathy is characterized by abnormal heart structure and dysfunction [2,3]. Increasing evidence suggests the protective effect of IF in obesity cardiomyopathy [28,29,30]; however, its molecular mechanism is not yet fully clarified. In the present study, we examined the therapeutic effect of IF on obesity cardiomyopathy as well as its impact on lipid deposition and m6A methylation in the heartof obese mice. Our study revealed that IF intervention protected against HFD-induced lipid deposition, apoptosis, and m6A methylation in cardiac tissues, as a result, restored cardiac functional and structural impairment. Mechanistically, IF may regulate myocardial lipid metabolism and apoptosis through m6A methylation in obesity cardiomyopathy. The present study, in part, shows that the protective effects of IF in cardiovascular protection may be involved in m6A RNA methylation.

Long-term HFD induces obese cardiomyopathy, leading to cardiac abnormal structure and dysfunction. Our results indicated that 8 weeks of IF intervention in obese mice could attenuate cardiac diastolic and systolic dysfunction, morphological abnormalities, and interstitial fibrosis. It was reported that the disorder of myocardial lipid metabolism is considered to be the major pathogenesis of obesity cardiomyopathy [3]. Previous studies have shown that serum lipid metabolism disorders in obese mice, such as increased levels of TC, TG, HDL, LDL, and FFA [31,32]. Furthermore, 24-week HFD resulted in increased cardiac TG content and LDs number and induced lipotoxic cardiomyopathy [33]. Similarly, in our results, abnormal serum lipid metabolism and cardiac lipid deposition were examined in obese mice but were ameliorated by IF intervention. Lipid synthesis and breakdown are highly regulated multistep reactions, with a pathway involving distinct sets of enzymatic reactions. Obesity induces the breakdown of the dynamic balance of myocardial lipid synthesis and decomposition and the abnormal transcription of the cardiac lipid accumulation, lipid synthesis, and catabolism genes [34]. We found that IF decreased the mRNA levels of fatty acid uptake genes of FABP1, FATP1, and CD36, the fatty acid synthesis genes of SREBF1, FAS, and ACCα, and increased the gene expression of fatty acid catabolism genes of ATGL, HSL, LAL, and LPL. Therefore, we speculated that IF alleviates HFD-induced cardiac lipid deposition by decreasing fatty acid uptake and synthesis and increasing fatty acid catabolism.

Myocardial lipid accumulation has been reported to promote myocardial apoptosis [35]. Previous research has shown that myocardial apoptosis increased in obese rats, as evidenced by an up-regulated TUNEL-positive cells, Bax/Bcl-2 ratio, and Cleaved Caspase-3 protein expression [36]. Another study reported that food restriction decreased TUNEL-positive cells, Bax/Bcl-2 ratio, and Cleaved Caspase-3 protein expression, which could inhibit cardiac apoptosis in HFD-induced obese rats [28]. Similarly, in our study, TUNEL staining and western blot analysis showed that cardiac lipid deposition significantly increased cardiac apoptotic cell death, and IF intervention markedly reversed HFD-induced these effects. In sum, IF appeared to inhibit HDF-induced cardiac apoptosis, but its underlying mechanism at the epitranscriptomic modification is not fully understood yet.

Growing evidence revealed that m6A methylation is related to obesity and lipid metabolic [37,38]. Previous study has shown that the mRNA and protein expression of FTO were upregulated in non-alcoholic fatty liver disease (NAFLD) rats, which were involved in lipid metabolism disorders [39]. The m6A methylation levels and METTL3 expression were raised in the livers of obese mice, and hepatocyte-specific knockout of METTL3 improved lipid metabolic disorders and insulin resistance [40]. A recent study showed that YTHDC2, an m6A reader, was significantly down-regulated in the livers of obese mice and NAFLD patients. Overexpression of YTHDC2 in the livers of obese mice improved hepatic lipid metabolism and insulin resistance [41]. In addition, increased m6A methylation levels were up-regulated in the impaired cardiovascular system and were involved in the pathogenesis of cardiovascular diseases, leading to cardiac apoptosis and dysfunction [20]. A recent study has revealed that m6A methylated levels were up-regulated and the gene and protein levels of FTO were reduced in human and mouse failing hearts, while FTO overexpression significantly decreased fibrosis and enhanced cardiomyocyte contractile function [42]. The m6A levels and METTL3 expression were up-regulated in hypoxia/reoxygenation (H/R)-treated cultured H9C2 cells and ischemia/reperfusion (I/R) mouse hearts. Cardiomyocyte-specific knockout of METTL3 promoted autophagic flux and reduced apoptosis in I/R mice [43]. Our study revealed that m6A methylation levels and METTL3 were consistently up-regulated, and FTO was consistently reduced in cardiac tissueof HFD-fed mice. It is worth noting that IF intervention markedly reversed HFD-induced these effects. We speculate that down-regulation the expression of METTL3 and up-regulation the expression of FTO may be responsible for altered m6A levels in the HFD-IF group. Additionally, it has been reported that IF can improve metabolic diseases such as obesity and aging by regulating epigenetic modifications [44,45]. Therefore, it is thought that IF may ameliorate HFD-induced obesity cardiomyopathy via decreasing m6A RNA methylation.

As indicated above, to the best of our knowledge, our study is the first to assess the effects of IF on m6A RNA methylation. Together, these results provide a novel pathological mechanism of HFD-induced obesity cardiomyopathy and suggest that reducing m6A methylation levels through intermittent fasting intervention is a therapeutic strategy for obesity-associated myocardial lipid deposition and apoptosis. Our study may help to design better non-pharmacological intervention protocols for obesity cardiomyopathy patients. However, one limitation of our manuscript is that it only detected the expression of m6A-associated genes and proteins by RT-PCR and western blot but did not conduct a genome-wide profiling of m6A-tagged transcripts in cardiac tissueby methylated RNA immunoprecipitation sequencing (MeRIP-Seq). Moreover, the molecular mechanism by which IF can attenuate cardiac lipid deposition and apoptosis via m6A methylation in obesity cardiomyopathy will need to be explored in further study.

## 5. Conclusions

**Figure 7:**
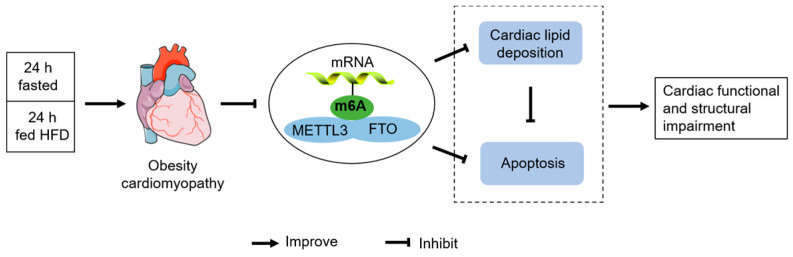
A possible mechanism for IF to improve obesity cardiomyopathy induced by HFD.

In summary, our results demonstrate that IF attenuated cardiac lipid deposition and apoptosis, as well as improved cardiac functional and structural impairment in HFD-induced obesity cardiomyopathy, by a mechanism associated with decreased m6A RNA methylation levels (Figure 7).

## Figures and Tables

**Table 1:** Primer Sequences Used for RT-PCR.

Genes	Primer Sequences	
	Forward (5′–3′)	Reverse (5′–3′)
FABP1	CCATGACTGGGGAAAAAGTC	GCCTTTGAAAGTTGTCACCAT
FATP1	TGCACAGCAGGTACTACCGCAT	TGCGCAGTACCACCGTCAAC
CD36	ATTGGTCAAGCCAGCT	TGTAGGCTCATCCACTAC
SREBP1c	AATCAGGACCATGCCG	CTCAACCTATGAAAATAAAGTTTGC
FAS	GCGGGTTCGTGAAACTGATAA	CAGGTTGGCATGGTTGACAG
ACCα	GCCTCCGTCAGCTCAGATAC	ATGTGAAAGGCCAAACCATC
ATGL	TGTTTCAGACGGAGAGAACG	GGAGGGGTGGAGGAATGAGG
LAL	TGGAGGGACAAACCACTGA	AAGGGAATCGGACCACTTG
HSL	CTTCTCCCTCTCGTCTGCTG	AATGGTCCTCTGCCTCTGTC
LPL	GATCCGAGTGAAAGCCGGAG	TTGTTTGTCCAGTGTCAGCCA
METTL3	CTGGGCACTTGGATTTAAGGAA	GTATCCCATCCAGTTGGTTTC
METTL14	CTGAGAGTGCGGATAGCATTG	GAGCAGATGTATCATAGGAAGCC
WTAP	TAGACCCAGCGATCAACTTGT	CCTGTTTGGCTATCAGGCGTA
FTO	TTCATGCTGGATGACCTCAATG	GCCAACTGACAGCGTTCTAAG
ALKBH5	GCATACGGCCTCAGGACATTA	TTCCAATCGCGGTGCATCTAA
YTHDF1	ACAGTTACCCCTCGATGAGTG	GGTAGTGAGATACGGGATGGGA
YTHDF2	GAGCAGAGACCAAAAGGTCAAG	CTGTGGGCTCAAGTAAGGTTC
YTHDF3	GATCAGCCTATGCCATATCTGAC	CCCCTGGTTGACTAAAAACACC
YTHDC1	GGAAGCACCCAGTGTATAGGA	GGAAGCACCCAGTGTATAGGA
YTHDC2	GAAGATCGCCGTCAACATCG	GCTCTTTCCGTACTGGTCAAA
GAPDH	GCAAGGACACTGAGCAAGA	GGATGGAAATTGTGAGGGAG

## Data Availability

The data presented in this study are available on request from the corresponding author.
